# Intrauterine Growth Restriction Affects Colonic Barrier Function *via* Regulating the Nrf2/Keap1 and TLR4-NF-κB/ERK Pathways and Altering Colonic Microbiome and Metabolome Homeostasis in Growing–Finishing Pigs

**DOI:** 10.3390/antiox13030283

**Published:** 2024-02-26

**Authors:** Liang Xiong, Md. Abul Kalam Azad, Yang Liu, Wanghong Zhang, Qian Zhu, Chengjun Hu, Jinming You, Xiangfeng Kong

**Affiliations:** 1 Key Laboratory of Agro-Ecological Processes in Subtropical Region, Hunan Provincial Key Laboratory of Animal Nutritional Physiology and Metabolic Process, National Engineering Laboratory for Pollution Control and Waste Utilization in Livestock and Poultry Production, Institute of Subtropical Agriculture, Chinese Academy of Sciences, Changsha 410125, China; 244628426@stu.scau.edu.cn (L.X.); azadmak@isa.ac.cn (M.A.K.A.); liuyang@stu.njau.edu.cn (Y.L.); 13616905519@stu.scau.edu.cn (W.Z.); zhuqian@isa.ac.cn (Q.Z.); yxh@stu.scau.edu.cn (C.H.); 2 Key Laboratory of Animal Nutrition in Jiangxi Province, College of Animal Science and Technology, Jiangxi Agricultural University, Nanchang 330045, China

**Keywords:** colonic microbiota, growing-finishing pigs, intrauterine growth restriction, metabolomics

## Abstract

Intrauterine growth restriction (IUGR) pigs are characterized by long-term growth failure, metabolic disorders, and intestinal microbiota imbalance. The characteristics of the negative effects of IUGR at different growth stages of pigs are still unclear. Therefore, this study explored through multi-omics analyses whether the IUGR damages the intestinal barrier function and alters the colonization and metabolic profiles of the colonic microbiota in growing-finishing pigs. Seventy-two piglets (36 IUGR and 36 NBW) were allocated for this trial to analyze physiological and plasma biochemical parameters, as well as oxidative damage and inflammatory response in the colon. Moreover, the colonic microbiota communities and metabolome were examined using 16s rRNA sequencing and metabolomics technologies to reveal the intestinal characteristics of IUGR pigs at different growth stages (25, 50, and 100 kg). IUGR altered the concentrations of plasma glucose, total protein, triglycerides, and cholesterol. Colonic tight junction proteins were markedly inhibited by IUGR. IUGR decreased plasma T-AOC, SOD, and GSH levels and colonic *SOD-1*, *SOD-2*, and *GPX-4* expressions by restraining the Nrf2/Keap1 signaling pathway. Moreover, IUGR increased colonic IL-1β and TNF-α levels while reducing IL-10, possibly through activating the TLR4-NF-κB/ERK pathway. Notably, IUGR pigs had lower colonic *Streptococcus* abundance and Firmicutes-to-Bacteroidetes ratio at the 25 kg BW stage while having higher Firmicutes abundance at the 100 kg BW stage; moreover, IUGR pigs had lower SCFA concentrations. Metabolomics analysis showed that IUGR increased colonic lipids and lipid-like molecules, organic acids and derivatives, and organoheterocyclic compounds concentrations and enriched three differential metabolic pathways, including linoleic acid, sphingolipid, and purine metabolisms throughout the trial. Collectively, IUGR altered the nutrient metabolism, redox status, and colonic microbiota community and metabolite profiles of pigs and continued to disrupt colonic barrier function by reducing antioxidant capacity *via* the Nrf2/Keap1 pathway and activating inflammation *via* the TLR4-NF-κB/ERK pathway during the growing-finishing stage. Moreover, colonic Firmicutes and *Streptococcus* could be potential regulatory targets for modulating the metabolism and health of IUGR pigs.

## 1. Introduction

Over the past few decades, genetic selection has gained a growing interest in increasing the litter size in swine production. However, the increase in litter size leads to a decrease in birth weight and an increase in the proportion of intrauterine growth restriction (IUGR) piglets [1]. IUGR refers to the impairment of the growth and development of the mammalian fetus or its organs during pregnancy. In modern swine production, the incidence rate of IUGR accounts for 15–20%, and approximately 75% of IUGR piglets die before weaning, resulting in a serious economic loss [2]. Therefore, it is essential to investigate the underlying mechanism of IUGR, which might assist in the development of strategies to prevent IUGR occurrence.

The mammalian hindgut, especially the colon, is colonized by numerous fermentative microbes. These microbes play fundamental roles in nutrient digestion and absorption, prevention of pathogenic colonization, and mucosal immunity regulation [3]. Recent studies indicated that IUGR exhibited lower abundances of anaerobic microbes, especially Lactobacilli and *Bifidobacterium*, and resulted in delaying early gut microbiota establishment [4]. Altered gut microbiota can induce changes in the metabolites. A recent study reported that IUGR altered the concentrations of fatty acids, lipids, and lipid-like molecules related to multiple metabolic pathways, including fatty acid metabolism and lipid biosynthesis [5]. Furthermore, microbiota synthesizes various compounds, including short-chain fatty acids (SCFAs), indoles, organic acids, and bioamines [6]. The sustaining alterations of the gut microbiota of IUGR piglets affect SCFA production, which might play a crucial role in long-term health consequences [7]. The balanced metabolic status of the gut microbiota is strongly associated with the health of the host. Therefore, compositional differences in the intestinal microbiome and metabolome profiles and their possible association with IUGR and normal birth weight (NBW) of growing-finishing pigs need to be further elucidated.

IUGR pigs are characterized by impaired gastrointestinal development, which further induces necrotizing colitis. Oxidative stress causes mucosal injury in the gastrointestinal tract, resulting in pathogenic invasions, which decompose and release lipopolysaccharide (LPS) and stimulate inflammatory and immune responses [8]. The gut microbes and metabolites affect the redox status of individuals. Previous studies indicated that several gut microbes, such as *Lactobacillus* and *Escherichia coli*, can synthesize catalase (CAT) to deactivate hydrogen peroxide and protect intestinal integrity [9,10]. The SCFAs stimulate glutathione-S-transferase and reduce oxidative stress [11]. Piglets experience oxidative stress at an early age, leading to a high risk of metabolic diseases later in life [12]. Previous research evidence showed that IUGR decreased intestinal glutathione (GSH) activity, indicating a lower antioxidant capacity in 21-day-old piglets [13]. Hence, investigating the effects of IUGR on the intestinal redox status of pigs during their lifelong development is vital for alleviating intestinal damage.

The changes in intestinal function, microbiota composition, and metabolic activity induced by IUGR persisted throughout the life of rats [7]. Our previous studies showed that IUGR altered the colonic metabolome and microbiome in pre-weaning piglets and reduced the abundances of Firmicutes, Proteobacteria, and *Lactobacillus* in growing-finishing pigs [14,15]. In addition, IUGR decreased the colonic expressions of zonula occludens (ZO)-1 and occludin, activated nuclear factor-kappa B (NF-κB), and increased inflammatory factor levels in pre-weaning piglets [15]. However, the effects of IUGR on the colonization and metabolic profiles of the colonic microbiota and the barrier function in growing-finishing pigs remain unclear. Therefore, we hypothesized that IUGR continued to damage the barrier function and alter the colonization and metabolic profiles of the colonic microbiota in growing-finishing pigs. Thus, the present study evaluated the long-term effects of IUGR on intestinal barrier function, microbiota colonization, and metabolic profiles in the colon, as well as the underlying mechanism in growing-finishing pigs. This study will provide a reference for improving the nutrient metabolism and gut homeostasis in IUGR pigs during the growing-finishing stage.

## 2. Materials and Methods

### 2.1. Animals, Experimental Design, and Diets

A total of thirty-six pregnant sows (Large White × Landrace) with similar body conditions were assigned for this study. After farrowing, the neonatal piglets were weighed promptly without colostrum intake. A total of 72 newborns (half male and half female) were selected from 36 litters, one IUGR piglet and one NBW piglet per litter. The piglets with the highest birth weight and the lowest (less than 1.0 kg) birth weight within the litter were defined as NBW and IUGR, respectively. Subsequently, selected piglets were ear-notched for identification. The cross-fostering within 24 h post-farrowing was not involved in these piglets. At weaning day (27 days of age), NBW and IUGR piglets were transferred to the individual nursery pens (1.2 × 0.8 m). After weaning, experimental piglets were fed individually. Pigs in the IUGR and NBW groups were fed the same basal nursery (during 28–69 days of age), growing (during 70–103 days of age), and finishing diets (during 104–165 days of age). Pigs in the NBW group reached the average body weight (BW) of 25, 50, and 100 kg at 69, 103, and 165 days of age, respectively. Any antibiotics were avoided during the entire trial. Feed and water were available *ad libitum*. The ingredients and nutrient levels of basal diets are presented in Table S1. These diets were designed to meet or exceed the nutrient reference for the National Research Council (NRC 2012).

### 2.2. Sample Collection

Twelve pigs from each group were selected to collect samples when the average body weight of the NBW pigs reached 25, 50, and 100 kg, respectively. Blood samples were collected through the precaval veins into 10 mL heparinized tubes, then centrifuged at 3000× *g* for 15 min at 4 °C to obtain plasma, and immediately stored at –20 °C for subsequent biochemical analyses. Pigs were euthanized by electrical stunning at 110 V and 2.4–2.8 A, followed by exsanguination. Colonic luminal contents (15 cm distally to the ileocecal valve) were collected and immediately stored at –80 °C for microbiome and metabolome analyses.

### 2.3. Laboratory Analysis

#### 2.3.1. Analysis of Plasma Biochemical Parameters

Plasma biochemical parameters, including albumin (ALB), ammonia (AMM), alanine aminotransferase (ALT), aspartate aminotransferase (AST), alkaline phosphatase (ALP), cholesterol (CHO), cholinesterase (CHE), globulin (GLB), glucose (GLU), high-density lipoprotein-cholesterol (HDL-C), total protein (TP), low-density lipoprotein-cholesterol (LDL-C), triglyceride (TG), and urea nitrogen (UN) were detected using the full-automatic biochemical analyzer (Roche, Basel, Switzerland) and available commercial reagent kits (Leadman Biochemistry Technology Company, Beijing, China) according to the manufacturer’s protocols.

#### 2.3.2. Analysis of Plasma Redox Status

The plasma total antioxidant capacity (T-AOC) and superoxide dismutase (SOD) activities and the levels of GSH, H_2_O_2_, and malondialdehyde (MDA) were detected using the commercially available assay kits (Comin Biotechnology, Suzhou, China) and read on a spectrophotometer (Tecan M200, Basel, Switzerland).

#### 2.3.3. Analysis of Colonic Redox Status and Inflammatory Cytokines

Colonic mucosal redox status biomarkers, including T-AOC, SOD, GSH, and MDA, were determined using the kits and instructions provided by Nanjing Jiancheng Biological Research Institute (Nanjing, China). Inflammatory cytokines, including interleukin (IL)-1β, IL-6, and tumor necrosis factor (TNF)-α, were detected using the porcine IL-1β ELISA Kit (ml002302), porcine IL-6 Kit (ml002311), and porcine TNF-α Kit (ml002360) (Enzyme-linked Biotechnology, Shanghai, China). Briefly, colonic mucosa samples were mixed with phosphate-buffered saline (1 g: 9 mL) and then homogenized at 3000× *g* and –20 °C for 15 min to break the cells. The total protein concentration of the colonic mucosa was detected using the BCA assay kit (Beyotime, Shanghai, China) following the kit protocols and read on a spectrophotometer. The measured inflammatory biomarkers of the colonic mucosa samples were normalized for calculation.

#### 2.3.4. Microbial DNA Isolation and 16S rRNA Gene Sequencing

The total microbial genomic DNA of colonic contents stored in EP tubes (Eppendorf, Hamburg, Germany) was extracted with the QiaAmp Fast DNA SPIN extraction kit (MP Biomedicals, Santa Ana, CA, USA) according to the manufacturer’s instructions. The concentrations of the extracted DNA were determined using a NanoDrop ND-1000 spectrophotometer (Thermo Fisher Scientific, Wilmington, MA, USA). The V3–V4 regions of microbial 16S rRNA genes were amplified using the forward primer 341F (5′-ACTCCTACGGGAGGCAGCAG-3′) and reverse primer 806R (5′-GGACTACHVGGGTWTCTAAT-3′). The PCR amplification reaction system was composed of 10.00 μL of Q5 reaction buffer (5×), 0.45 μL of Q5 FastPfu polymerase (5 μM), 2.00 μL of 2.50 mM deoxynucleoside triphosphates, 1.00 μL (5 μM) of each forward and reverse primers, 2.00 μL of cDNA template, and 8.55 μL of ddH_2_O. The PCR amplification reactions program consisted of a thermal cycle as follows: 3 min of denaturation at 95 °C; 25 cycles of 30 s at 98 °C, 30 s of annealing at 55 °C, and extension at 72 °C for 30 s; and a final extension at 72 °C for 10 min. The purified PCR amplicon products were pooled into an equimolar mixture and subjected to pair-end (2 × 300 bp) sequencing on an Illumina MiSeq 2500 platform library (Illumina, San Diego, CA, USA) following the standard protocols by the Shanghai Personal Biotechnology Co., Ltd. (Shanghai, China).

The alpha diversity of the operational taxonomic unit (OTU) level was analyzed using the QIIME software (version 2.0). Beta diversity analysis was performed to investigate the structural variations of the microbial community between the NBW and IUGR groups by the principal component analysis (PCA) dependent on unweighted UniFrac distance. Partial least squares discriminant analysis (PLS-DA) dependent on unweighted UniFrac distances was further performed as a supervised model to exhibit the bacterial differences between the two groups by the PLS-DA program in R package “mixOmics” as previously described [14]. The phylogenetic investigation of the microbial communities by reconstruction of unobserved states (PICRUSt) analysis was used to predict the microbial gene function dependent on the Kyoto Encyclopedia of Genes and Genomes (KEGG) pathways.

#### 2.3.5. Colonic Metabolite Extraction and Non-Targeted Metabolomics Analysis

A total of 60 colonic contents (*n* = 10) were detected using an ultra-high-performance liquid chromatography-mass spectrometry (UPLC-MS) platform. Approximately 25 mg sample was transferred into a 1.5 mL centrifuge tube (Corning, NY, USA) and mixed with 500 μL extract solution (the ratio of acetonitrile to methanol to water was 2:2:1, containing isotopically labeled internal standard mixture).

UPLC-MS analyses were performed using a UHPLC system coupled with a Q-Exactive mass spectrophotometer (Thermo Fisher Scientific, Waltham, MA, USA). The mobile phases were divided into positive and negative ion modes. For positive ion mode, the mobile phase A is composed of water (adjusted pH = 9.75) with formic acid (water: formic acid; 2:1), and the mobile phase B is composed of acetonitrile with 0.1% formic acid (acetonitrile: formic acid; 3:1). For negative ion mode, 25 mM ammonium acetate and 25 mM ammonia hydroxide in water (pH = 9.75) replaced the formic acid in mobile phase A and B. The injecting volume of the automatic sampler was 3.00 μL at 4 °C. Using the data acquisition software Xcalibur version 4.1 (Thermo Fisher Scientific, Waltham, MA, USA), the QExactive HFX mass spectrometry was performed to obtain the MS spectrum on information-dependent acquisition (IDA) mode. Under this mode, the acquisition software serially assessed the full scan MS spectra. The electrospray ionization (ESI) source conditions were set as follows: sheath gas flow rate of 30 Arb, aux gas flow rate of 25 Arb, capillary temperature of 350 °C, full MS resolution of 60,000, and MS/MS resolution of 7500, respectively. The raw data were converted into mzXML format using the ProteoWizard and processed using an XCMs-based internal program developed by the R studio for peak detection, extraction, alignment, and integration. Finally, differential metabolite annotation was performed with the internal MS2 database (BiotreeDB). The cut-off value for annotation was set to 0.30.

#### 2.3.6. Determination of SCFAs, Indole, Skatole, and Bioamines in Colonic Contents

Colonic contents (~1.00 g) were weighed and mixed with 5.00 mL ultrapure water and then centrifuged at 1000× *g* and 4 °C for 10 min to collect supernatants. The obtained supernatants were mixed with 25% metaphosphoric acid solution to determine the concentrations of SCFAs, including straight-chain fatty acids (acetate, butyrate, propionate, and valerate) and branched-chain fatty acids (BCFAs; isobutyrate and isovalerate) using the gas chromatography (Agilent Technologies Inc., Palo Alto, CA, USA) as previously described [15].

Approximately 100 mg of colonic contents were weighed and mixed with 1.00 mL of acetonitrile. Colonic contents mixtures were vortexed and then centrifuged at 3000× *g* for 10 min at 4 °C to collect supernatants. After filtering through a 0.22-µm membrane, supernatants were used to determine the concentrations of indole, skatole, and bioamines using high-performance liquid chromatography (Agilent Technologies, Palo Alto, CA, USA). The determined bioamines included cadaverine, phenylethylamine, putrescine, spermidine, spermine, tryptamine, tyramine, and 1,7-heptyl diamine [16].

#### 2.3.7. Analysis of Oxidative and Inflammatory-Related Genes

The mRNA expressions of glutathione peroxidase 1 (*GPX-1*), *GPX-4*, *SOD-1*, *SOD-2*, *IL-1β*, *IL-10*, and *TNF-α* were determined by RT-qPCR. Briefly, the total RNA was extracted from colonic mucosa tissues with the TRIzol reagent (Invitrogen, Carlsbad, CA, USA) according to the manufacturer’s protocols. The concentration of the extracted RNA was detected using a NanoDrop ND-1000 spectrophotometer (NanoDrop Technologies Inc., Wilmington, DE, USA), and the quality was determined using the ratio of A260/A280. The total RNA was reverse-transcribed into cDNA using the Prime Script RT Reagent Kit with gDNA Eraser (Takara, Dalian, China) for quantitative PCR analysis. The primers of the target genes and reference gene β-actin are listed in Table S2. An RT-PCR analysis was performed on the Light Cycler^®^ 480 II Real-Time PCR System (Roche, Basel, Switzerland). The PCR cycle conditions were set as follows: 1 cycle denaturation at 95 °C for 5 min, followed by 40 cycles of 95 °C for 5 s, and annealing at 60 °C for 35 s. Relative mRNA expressions were calculated using the 2^−ΔΔCt^ method and were normalized to β-actin level [16].

#### 2.3.8. Analysis of the Relative Protein Abundances

Equal amounts of protein were extracted from colonic mucosa by RIPA lysate buffer (Beyotime, Shanghai, China), which contained 1% protease inhibitors phenylmethyl sulfonyl fluoride (PMSF) and 1% phosphatase inhibitors cocktail. The total protein concentration was measured using the BCA assay kit (Beyotime, Shanghai, China). Resolution of protein was determined via sodium dodecyl sulfate-polyacrylamide gel electrophoresis (SDS-PAGE) gel (Genscript, Nanjing, China), followed by a transfer onto polyvinylidene difluoride (PVDF) membranes at 140 V for 45 min. The membranes were nonspecifically blocked with 5% skim milk buffer for 1 h. The membranes were incubated overnight with primary antibodies against β-actin (#bs-0061R, Bioss), Claudin1 (#ab211737, Abcam), Occludin (#ab216327, Abcam), Zonula Occludens (ZO)-1 (#21773-1-AP, Proteintech), TLR4 (#14358, CST), NF-κB (#ab32536, Abcam), p-NF-κB (#3033, CST), ERK1/2 (#9194, CST), p-ERK1/2 (#4370, CST), Nrf2 (#bs-1074R, Bioss), p-Nrf2 (bs-2013R, Bioss), and Keap1 (#bs-3648R, Bioss) at 4 °C. After that, membranes were washed three times with TBST (phosphate buffered saline with Twen-20) buffer, and then incubated with a suitable horseradish peroxidase coupled secondary antibodies (HRP-conjugated Goat Anti-Rabbit IgG, Proteintech, Wuhan, China) for 1.5 h at room temperature. Finally, the protein expression in immunoreactive target bands was visualized using the FluorChem M (ProteinSimple, San Jose, CA, USA). The ImageJ software version 1.8.0 (National Institutes of Health, Bethesda, MD, USA) was performed to analyze the gray scan value normalized against β-actin.

### 2.4. Statistical Analysis

The comparative analyses for the plasma parameters, colonic metabolites, protein abundances, and mRNA expressions were assessed using the SPSS 22.0 (Chicago, IL, USA) software and Student’s *t*-test. Data are expressed as means ± standard error of the mean (SEM). *p*-values < 0.05 were considered significant differences. The microbial alpha diversity and relative abundances were analyzed using the Mann-Whitney *U*-test. The linear discriminant analysis (LDA) effect size (LEfSe) was conducted using the Wilcoxon rank-sum test. The Wilcoxon rank-sum test with histograms of the LDA score was used to detect abundant differential taxa using the default parameters between NBW and IUGR pigs. Wilcoxon rank-sum test with FDR correction was performed to detect the significantly different KEGG pathways (level 3) between NBW and IUGR pigs using the STAMP software (version 2.1.3). The correlations between colonic metabolites and microbiota were measured using Spearman’s correlation analysis by the R package ggplot2 (version 3.3.1). GraphPad Prism V.6.0 (San Diego, CA, USA) was used to plot the images. The multivariate and statistical analyses were performed using MetaboAnalyst 4.0 for metabolome data. The PCA, PLS-DA, and *t*-test were performed with the FDR adjusted *p*-value < 0.05, and the variable importance in projection (VIP) > 1 was considered significantly differential metabolites.

## 3. Results

### 3.1. Effects of IUGR on Plasma Biochemical Parameters in Growing-Finishing Pigs

The effects of IUGR on plasma biochemical parameters are presented in Table 1. Compared with the NBW pigs, IUGR pigs had higher (*p* < 0.05) levels of ALP at the 25 kg BW stage, AMM, ALT, CHO, and TG at the 50 kg BW stage, and UN at the 100 kg BW stage. In addition, IUGR pigs had lower (*p* < 0.05) levels of GLU at the 25 kg BW stage, ALB, GLU, TP, and CHE at the 50 kg BW stage, and ALB and TP at the 100 kg BW stage compared with the NBW pigs.

### 3.2. Effects of IUGR on Plasma Redox Status in Growing-Finishing Pigs

**Figure 1:**
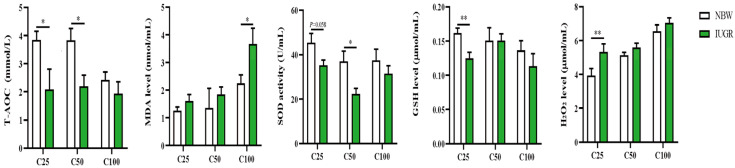
Effects of intrauterine growth restriction (IUGR) on plasma redox status in growing-finishing pigs (*n* = 10). * *p* < 0.05, ** *p* < 0.01. C25, C50, and C100 represent the plasma samples obtained from the pigs when the normal birth weight (NBW) pigs reached 25, 50, and 100 kg body weight. GSH, glutathione; MDA, malondialdehyde; SOD, superoxide dismutase; T-AOC, total antioxidant capacity.

The plasma redox status between IUGR and NBW pigs is shown in Figure 1. The plasma T-AOC and GSH levels at the 25 kg BW stage and T-AOC and SOD activity at the 50 kg BW stage were lower (*p* < 0.05) in the IUGR pigs than those in the NBW pigs. The MDA level was higher (*p* < 0.05) in the IUGR pigs than in the NBW pigs at the 100 kg BW stage.

### 3.3. Effects of IUGR on Colonic Tight Junction Protein Abundances in Growing-Finishing Pigs

**Figure 2:**
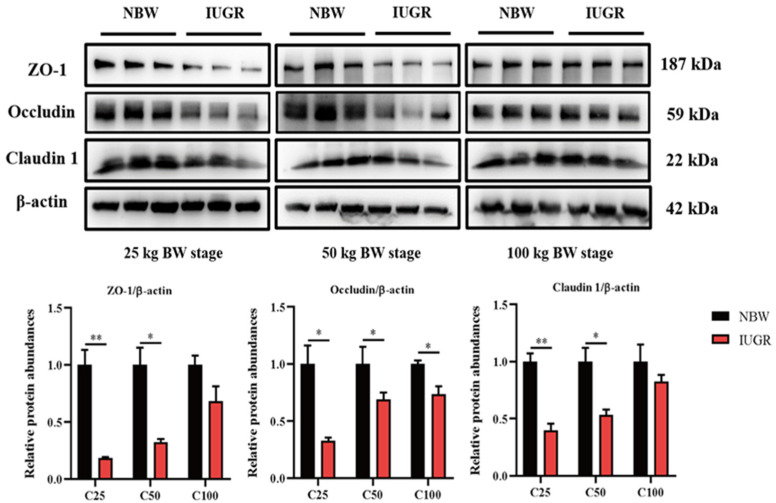
Effects of intrauterine growth restriction (IUGR) on the colon mucosal tight junction proteins in growing-finishing pigs (*n* = 6). * *p* < 0.05, ** *p* < 0.01. C25, C50, and C100 represent the samples obtained from the colonic mucosa of pigs when the normal birth weight (NBW) pigs reached 25, 50, and 100 kg body weight. ZO-1, zonula occludens.

The abundances of colonic tight junction protein between IUGR and NBW pigs are shown in Figure 2. The abundances of occludin in the three BW stages and claudin1 and ZO-1 at the 25 and 50 kg BW stages in IUGR pigs were lower (*p* < 0.05) than those in the NBW pigs.

### 3.4. Effects of IUGR on Colonic Mucosal Redox Status and Inflammatory Cytokines in Growing-Finishing Pigs

**Figure 3:**
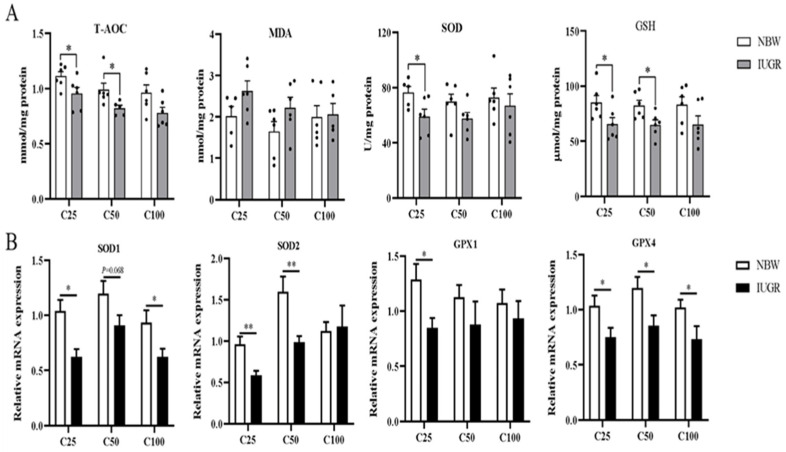
Effects of intrauterine growth restriction (IUGR) on the levels (**A**) and relative mRNA expressions (**B**) of colonic mucosal oxidative status parameters in growing-finishing pigs (*n* = 10). * *p* < 0.05, ** *p* < 0.01. C25, C50, and C100 represent the samples obtained from the colon mucosa of pigs when the normal birth weight (NBW) pigs reached 25, 50, and 100 kg body weight. T-AOC, total antioxidant capacity; MDA, malondialdehyde; SOD, superoxide dismutase; GSH, glutathione; GPX, glutathione peroxidase.

**Figure 4:**
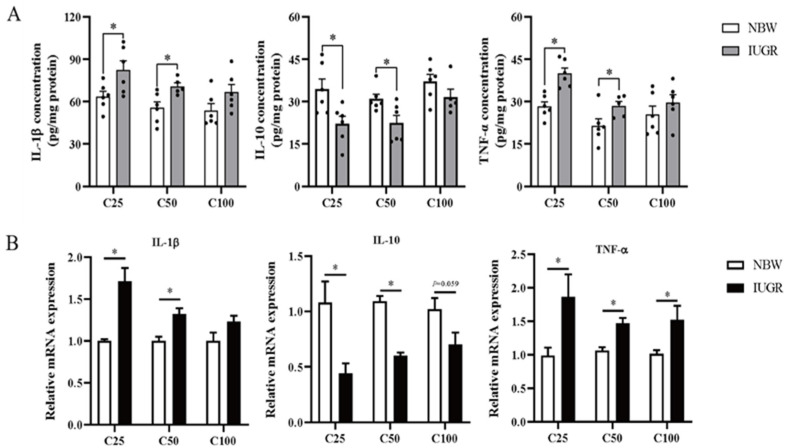
Effects of intrauterine growth restriction (IUGR) on the levels (**A**) and relative mRNA expressions (**B**) of colonic mucosal inflammatory cytokines in growing-finishing pigs (*n* = 10). * *p* < 0.05. C25, C50, and C100 represent the samples obtained from the colonic mucosa of pigs when the normal birth weight (NBW) pigs reached 25, 50, and 100 kg body weight. *IL-1β*, interleukin-1β; *IL*-10, interleukin 10; *TNF-α*, tumor necrosis factor-α.

The oxidative parameters and inflammatory cytokines in the colonic mucosa of IUGR and NBW pigs are shown in Figure 3A and Figure 4A, respectively. Compared with the NBW pigs, the levels of IL-1β and TNF-α were higher, while T-AOC and GSH concentrations and IL-10 level were lower in the IUGR pigs at the 25 and 50 kg BW stages (*p* < 0.05). The SOD activity was lower (*p* < 0.05) in the IUGR pigs at the 25 kg BW stage compared with the IUGR pigs. There was no significant difference (*p* > 0.05) in the colonic concentration of MDA and inflammatory cytokines at the 100 kg BW stage between NBW and IUGR pigs.

### 3.5. Effects of IUGR on Colonic Mucosal Oxidative and Inflammatory-Related mRNA Expressions in Growing-Finishing Pigs

The effects of IUGR on colonic mucosal oxidative and inflammatory-related gene expressions are shown in Figure 3B and Figure 4B. Compared with the NBW pigs, colonic *IL-10*, *SOD-1*, and *GPX-4* expressions were down-regulated, but *TNF-α* expression was up-regulated in the IUGR pigs at the three BW stages (*p* < 0.05). Moreover, the *SOD-2* expression was down-regulated, while *IL-1β* expression was up-regulated in the IUGR pigs at the 25 and 50 kg BW stages compared with the NBW pigs (*p* < 0.05). The *GPX-1* expression was down-regulated (*p* < 0.05) in the IUGR pigs than in the NBW pigs at the 25 kg BW stage.

### 3.6. Effects of IUGR on Colonic Mucosal Nrf2/Keap1 and TLR4-NF-κB/ERK Pathways in Growing-Finishing Pigs

**Figure 5:**
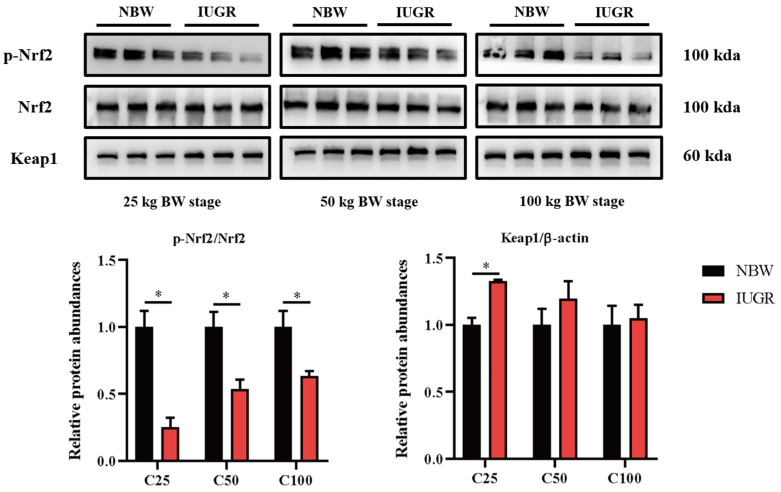
Effects of intrauterine growth restriction (IUGR) on colonic Nrf2/Keap1 signaling pathway in growing-finishing pigs (*n* = 6). * *p* < 0.05. C25, C50, and C100 represent the samples obtained from the colonic mucosa of pigs when the normal birth weight (NBW) pigs reached 25, 50, and 100 kg body weight.

**Figure 6:**
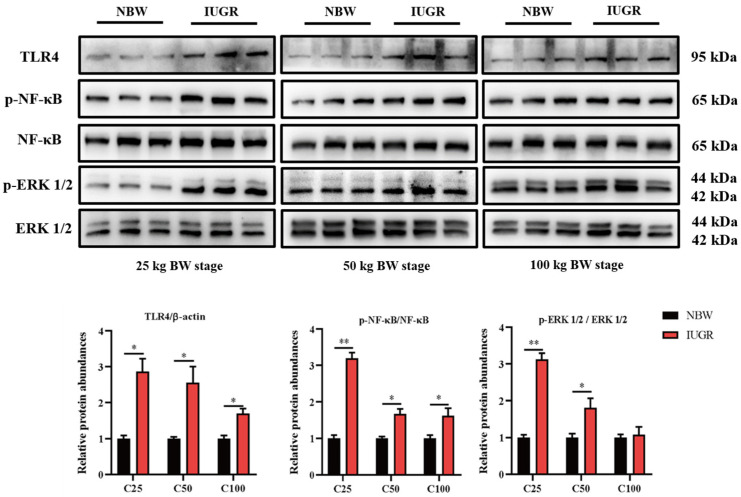
Effects of intrauterine growth restriction (IUGR) on colonic TLR4-NF-κB/ERK signaling pathway in growing-finishing pigs (*n* = 6). * *p* < 0.05, ** *p* < 0.01. C25, C50, and C100 represent the samples obtained from the colonic mucosa of pigs when the normal birth weight (NBW) pigs reached 25, 50, and 100 kg body weight.

To investigate the effects of IUGR on the intestinal barrier function further, Western Blot analysis was performed to detect the pathways related to antioxidants (Figure 5) and inflammation (Figure 6). The Nrf2 phosphorylation was lower (*p* < 0.05) at the three BW stages, and Keap1 abundance was higher (*p* < 0.05) in the IUGR pigs at the 25 kg BW stage than those in the NBW pigs (Figure 5). The TLR4 abundance and NF-κB phosphorylation were higher in the IUGR pigs at the three BW stages compared with the NBW pigs (*p* < 0.05). Furthermore, the ERK1/2 phosphorylation was higher (*p* < 0.05) in the IUGR pigs at the 25 and 50 kg BW stages than in the NBW pigs (Figure 6).

### 3.7. Effects of IUGR on Colonic Microbial Diversity in Growing-Finishing Pigs

**Figure 7:**
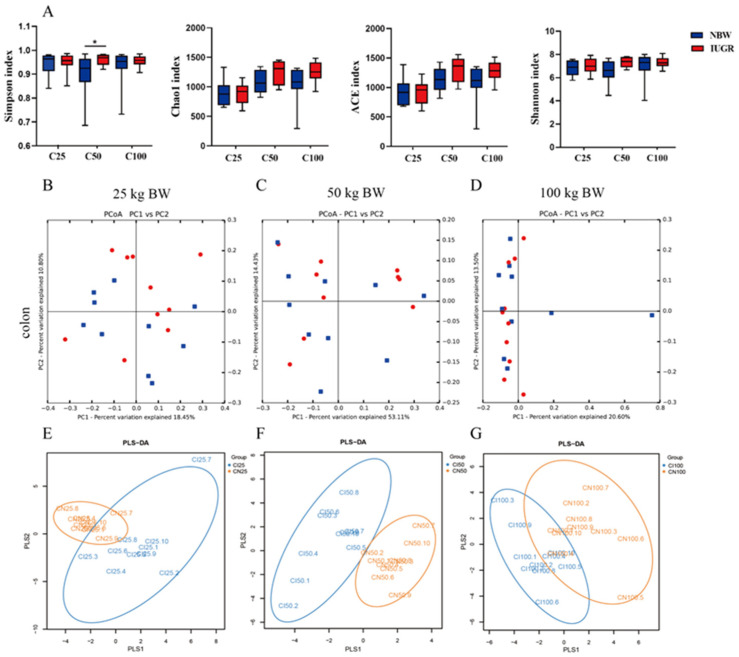
Differences in microbial alpha-diversity in colonic contents between the intrauterine growth restriction (IUGR) pigs and normal birth weight (NBW) pigs (**A**). * *p* < 0.05. Scatterplots from the principal component analysis (PCA) (**B**–**D**) and partial least square discriminant analysis (PLS-DA) (**E**–**G**) of OTUs show the differences in microbial community structures (*n* = 10). Each symbol represents the colonic microbiota of one pig (● IUGR; ■ NBW). C25, C50, and C100 represent the samples obtained from the colon of pigs when the NBW pigs reached 25, 50, and 100 kg body weight.

A total of 203,382 high-quality sequences were obtained from 60 colonic samples at the 25, 50, and 100 kg BW stages. Samples were randomly subsampled to 27,223 sequences to avoid bias caused by different sequencing depths. Based on 97% similarity, 4018 OTUs were obtained. Rarefaction curves indicated that almost all bacterial species were captured from colonic samples (Figure S1). As shown in Figure 7A, IUGR pigs had a higher (*p* < 0.05) Simpson index than the NBW pigs at the 50 kg BW stage. The PCA showed no distinct separation between the NBW and IUGR pigs (Figure 7B–D), and PLS-DA analysis showed a clear separation and assembled into two groups at the 50 kg BW stage (Figure 7E–G).

### 3.8. Effects of IUGR on Colonic Microbial Composition in Growing-Finishing Pigs

**Figure 8:**
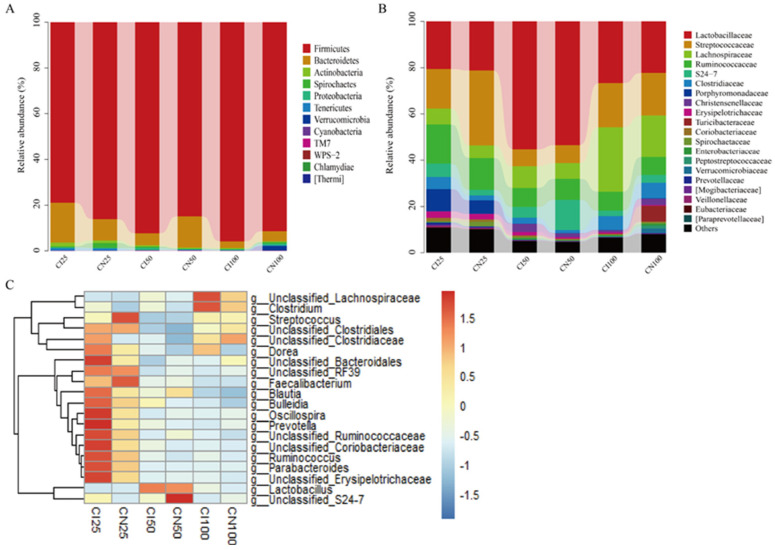
Colonic microbiota composition of intrauterine growth restriction (IUGR) pigs and normal birth weight (NBW) pigs at the 25, 50, and 100 kg body weight (BW) stages at the phylum (**A**), family (**B**), and genus (**C**) levels. The top 20 abundant genera with a proportion of >0.01 are listed. CI and CN represent the samples obtained from the colon of IUGR pigs and NBW pigs, respectively; 25, 50, and 100 represent 25, 50, and 100 kg BW stages, respectively.

The microbial community analysis for all samples was performed between the IUGR and NBW groups at the phylum, family, and genus levels (Figure 8). A total of 12 phyla, 54 families, and 95 genera were identified in the colon of IUGR and NBW pigs.

At the phylum level (Figure 8A), the top three dominant phyla were Firmicutes (NBW 86.07% vs. IUGR 78.91%), Bacteroidetes (9.42% vs. 17.50%), and Actinobacteria (1.10% vs. 1.63%) at the 25 kg BW stage; Firmicutes (84.85% vs. 92.37%), Bacteroidetes (13.75% vs. 5.21%), and Proteobacteria (0.29% vs. 1.01%) at the 50 kg BW stage; and Firmicutes (91.42% vs. 95.86%), Bacteroidetes (4.43% vs. 2.90%), and Proteobacteria (0.85% vs. 0.40%) at the 100 kg BW stage, respectively.

At the family level (Figure 8B), the top three dominant families were Streptococcaceae (NBW 32.44% vs. IUGR 17.15%), Lactobacillaceae (21.13% vs. 20.51%), and Ruminococcaceae (13.65% vs. 16.92%) at the 25 kg BW stage; Lactobacillaceae (53.45% vs. 55.23%), Ruminococcaceae (9.06% vs. 8.15%), and Streptococcaceae (7.83% vs. 7.40%) at the 50 kg BW stage; and Lactobacillaceae (22.21% vs. 26.71%), Lachnospiraceae (27.88% vs. 17.97%), and Streptococcaceae (18.47% vs. 19.04%) at the 100 kg BW stage, respectively.

The distribution of the colonic microbiota at the genus level is shown in Figure 8C. The top four genera in the NBW and IUGR pigs were *Lactobacillus* (NBW 21.13% vs. IUGR 20.51%), *Streptococcus* (32.25% vs. 16.83%), *unclassified_Ruminococcaceae* (10.25% vs. 12.37%), *unclassified_Clostridiales* (NBW 7.76%), and *Parabacteroides* (IUGR 9.50%) at the 25 kg BW stage; *Lactobacillus* (53.45% vs. 55.23%), *unclassified_Ruminococcaceae* (8.19% vs. 7.35%), *Streptococcus* (7.48% vs. 6.97%), *unclassified_Lachnospiraceae* (IUGR 6.98%), and *unclassified_S24-7* (NBW 13.11%) at the 50 kg BW stage; and *Lactobacillus* (22.21% vs. 26.71%), *unclassified_Lachnospiraceae* (16.26% vs. 25.26%), *Streptococcus* (17.96% vs. 18.48%), and *unclassified_Ruminococcaceae* (6.98% vs. 7.43%) at the 100 kg BW stage, respectively.

### 3.9. Effects of IUGR on the Taxonomic Differences in Colonic Microbiota in Growing-Finishing Pigs

The taxonomic differences in the colonic microbiota of IUGR and NBW pigs are shown in Table 2. The Firmicutes-to-Bacteroidetes (F/B) ratio and *Streptococcus* abundance were lower (*p* < 0.05) at the 25 kg BW stage, whereas Lactobacillaceae abundance at the 25 kg BW stage and Firmicutes abundance at the 100 kg BW stage were higher (*p* < 0.05) in the IUGR pigs than those in the NBW pigs.

**Figure 9:**
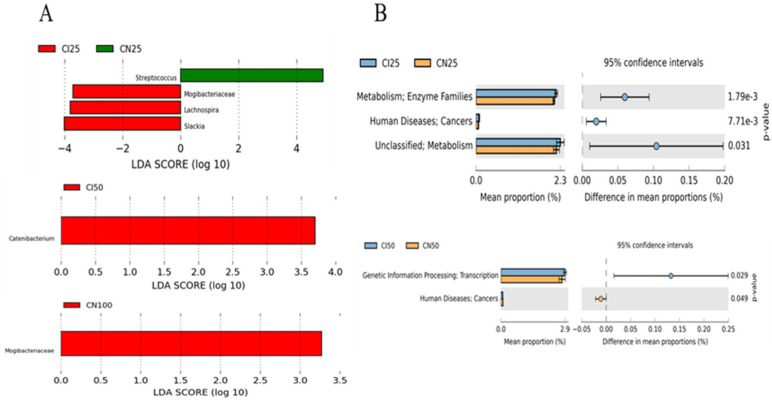
LEfSe analysis (**A**) at the genus level (LDA score ≥ 2) and PICRUSt analysis (level 2) (**B**) of predictive metagenomics function of colonic microbial community between intrauterine growth restriction (IUGR) pigs and normal birth weight (NBW) pigs at the 25, 50, and 100 kg body weight (BW) stages. CI and CN represent samples obtained from the colon of IUGR pigs and NBW pigs, respectively; 25, 50, and 100 represent 25, 50, and 100 kg BW stages, respectively.

Furthermore, the top 50 abundant genera of the colonic microbiota were determined using the LEfSe analysis (Figure 9A). The results showed that *Streptococcus* abundance was higher (*p* < 0.05), while *Mogibacteriaceae*, *Lachnospira*, and *Slackia* abundances were lower (*p* < 0.05) in the IUGR pigs than those in the NBW pigs at the 25 kg BW stage. *Catenibacterium* and *Mogibacteriaceae* abundances were higher (*p* < 0.05) in the IUGR pigs than in the NBW pigs at the 50 kg and 100 kg BW stages, respectively.

### 3.10. Effects of IUGR on Colonic Microbial Gene Functions in Growing-Finishing Pigs

The PICRUSt analysis was performed to predict colonic gene functions in growing-finishing pigs (Figure 9B). The enzyme families, cancers, and metabolism pathways were enriched in the IUGR pigs at the 25 kg BW stage. Furthermore, the transcription pathway related to genetic information processing was enriched in the IUGR pigs, whereas the pathway related to cancers was enriched in the NBW pigs at the 50 kg BW stage. However, there was no pathway enrichment at the 100 kg BW stage.

### 3.11. Effects of IUGR on the Concentrations of SCFAs, Indole, Skatole, and Bioamines in Colonic Contents of Growing-Finishing Pigs

The effects of IUGR on colonic SCFA concentrations are presented in Table 3. The colonic isobutyrate, butyrate, isovalerate, and BCFAs concentrations were lower (*p* < 0.05) in the IUGR pigs at the 25 kg BW stage; butyrate and valerate concentrations were lower (*p* < 0.05) in the IUGR pigs at the 50 kg BW stage; and acetate, isobutyrate, and BCFAs concentrations were lower (*p* < 0.05) in the IUGR pigs at the 100 kg BW stage, when compared with the NBW pigs.

As shown in Table 4, colonic cadaverine concentration was higher (*p* < 0.05) at the 25 kg BW stage, while colonic indole and putrescine concentrations at the 50 and 100 kg BW stages and cadaverine concentration at the 100 kg BW stage were lower (*p* < 0.05) in the IUGR pigs than those in the NBW pigs.

### 3.12. Correlation between Colonic SCFAs, Indole, Skatole, and Bioamines Concentrations and Microbiota Abundances

Spearman’s correlation matrixes were generated to explore the correlation between colonic metabolite concentrations and the top 20 abundant taxa at the genus level at different BW stages (Figure S2).

### 3.13. Effects of IUGR on Colonic Metabolome Profiles in Growing-Finishing Pigs

**Figure 10:**
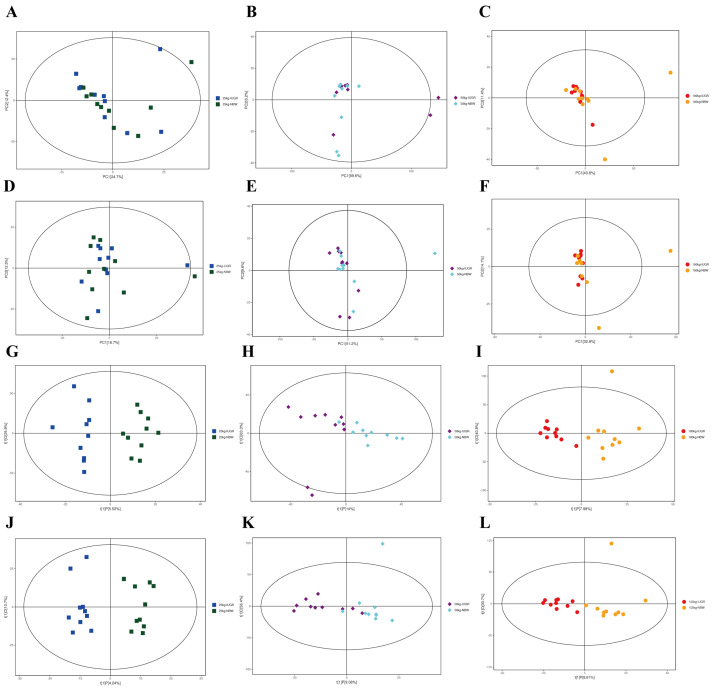
Score plots of principal component analysis (PCA) (**A**–**F**) and orthogonal partial least square discriminant analysis (OPLS-DA) (**G**–**L**) model derived from the UPLC–(+) ESI–MS/MS data of colonic metabolites of intrauterine growth restriction (IUGR) pigs and normal birth weight (NBW) pigs at the 25, 50, and 100 kg body weight (BW) stages. (**A**–**C**) represent PCA in ESI^+^ at the 25, 50, and 100 kg BW stages, respectively; (**D**–**F**) represent PCA in ESI^–^ at the 25, 50, and 100 kg BW stages, respectively; (**G**–**I**) represent OPLS-DA in ESI^+^ at the 25, 50, and 100 kg BW stages, respectively; (**J**–**L**) represent OPLD-DA in ESI^–^ at the 25, 50, and 100 kg BW stages, respectively.

The results analyzed by UPLC-QE-MS based on the non-target metabolomics showed that the PCA score plots did not show a clear separation (Figure 10A–F); however, OPLS-DA showed a clear separation in positive and negative ion modes between NBW and IUGR pigs at three BW stages (Figure 10G–L).

Overall, a total of 603 compounds were identified in the colonic metabolome. After filtering, 45 metabolites had significant differences (fold change > 1.5 or <1.0, VIP > 1). Compared with the NBW pigs, eight colonic differential metabolites, including phosphatidyl ethanolamine (PE), O-propanoyl-carnitine, (R)-pelletierine, N-a-acetyl-L-arginine, questiomycin A, 12,13-EpOME, (2S,4R)-4-(9H-pyrido[3,4-b]indol-1-yl)-1,2,4-butanetriol, and squamolone were increased (*p* < 0.05), whereas phytosphingosine was decreased (*p* < 0.05) in the IUGR pigs at the 25 kg BW stage. At the 50 kg BW stage, colonic concentrations of lupulone and phosphatidylcholines (PC) were increased (*p* < 0.05) in the IUGR pigs compared with the NBW pigs. At the 100 kg BW stage, 34 colonic differential metabolites, including 5-pyridoxolactone, histidinal, 4-pyridoxic acid, palmitoyl serinol, deoxyadenosine, deoxycytidine, and others were increased (*p* < 0.05) in the IUGR pigs compared with the NBW pigs. Notably, the colonic concentrations of pyridoxolactone, histidinal, and pyridoxic acid in the IUGR pigs had 5-, 4-, and 4-fold increases, respectively.

**Figure 11:**
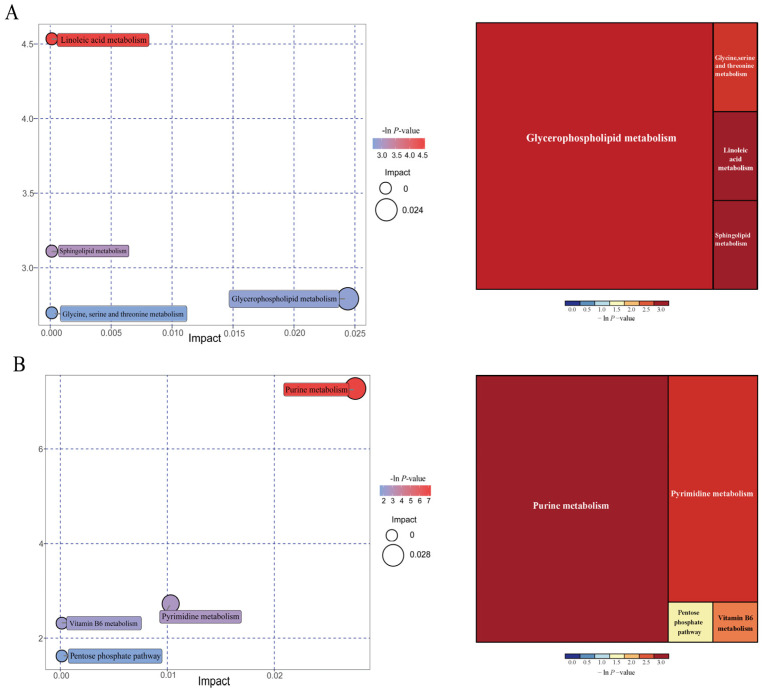
Pathway analysis of the colonic metabolites in the intrauterine growth restriction (IUGR) pigs and normal birth weight (NBW) pigs at the 25 (**A**) and 100 (**B**) kg body weight (BW) stages. The *X*-axis represents the impact factors of the pathway in topological analysis, and the *Y*-axis represents the *p*-value in pathway enrichment.

As shown in Figure 11, further metabolite enrichment analysis indicated that the differential metabolites between IUGR and NBW pigs were mapped into four metabolic pathways, including glycerophospholipid metabolism, linoleic acid metabolism, sphingolipid metabolism, and glycine/serine/threonine metabolism at the 25 kg BW stage (Figure 11A), and four metabolic pathways, including purine metabolism, pyrimidine metabolism, vitamin B_6_ metabolism, and pentose phosphate pathway at the 100 kg BW stage (Figure 11B). There were no significantly enriched metabolic pathways at the 50 kg BW stage. These metabolism pathways included seven significantly differential metabolites: 12,13-EpOME, phytosphingosine, deoxyadenosine, guanine, deoxyguanosine, guanosine, and adenine (Table 5).

### 3.14. Correlations between Colonic Microbiota Abundance and Differential Metabolite Concentrations of NBW and IUGR Pigs

**Figure 12:**
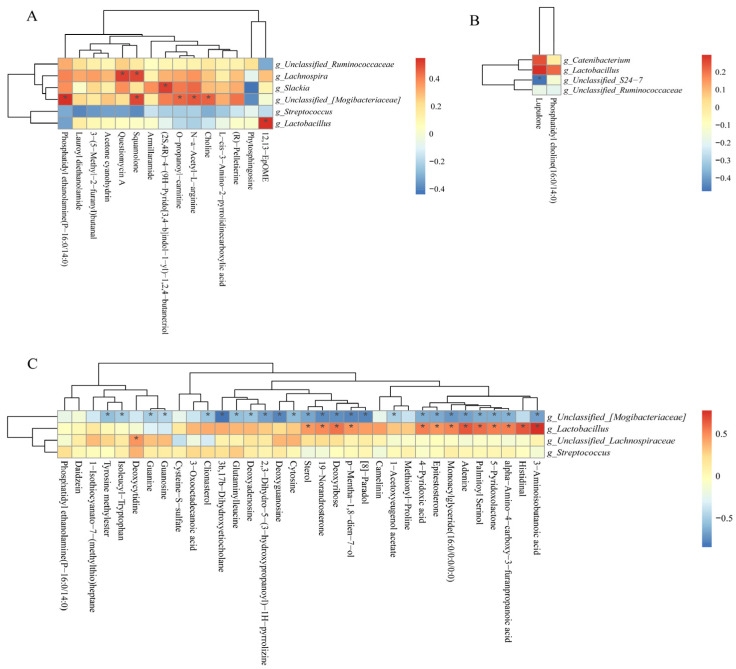
Spearman correlation analysis of differential microbial genera and potential differential metabolites (fold change > 1.5 or <1.0, VIP > 1.0) at the 25 (**A**), 50 (**B**), and 100 (**C**) kg body weight stages. * indicates significant correlations between intrauterine growth restriction (IUGR) pigs and normal birth weight (NBW) pigs; the red color represents a positive correlation, and the blue color represents a negative correlation.

As shown in Figure 12A, the positive correlation (*p* < 0.05) included between *Lachnospira* with questiomycin A and squamolone; *unclassified_[Mogibacteriaceae]* with PE, squamolone, O-propanoyl-carnitine, choline, and N-a-acetyl-L-arginine; *Slackia* and *Lactobacillus* with (2S,4R)-4-(9H-pyrido[3,4-b]indol-1-yl)-1,2,4-butanetrio and 12,13-EpOME at the 25 kg stage. As shown in Figure 12B, the negative correlation (*p* < 0.05) included between *unclassified_S24-7* with lupulone and *unclassified_[Mogibacteriaceae]* with 25 differential metabolites (Figure 12C) at the 50 and 100 kg BW stages, respectively. Furthermore, the positive correlation (*p* < 0.05) included *Lactobacillus* with 13 differential metabolites, as well as *unclassified_Lachnospiraceae* with deoxycytidine at the 100 kg BW stage (Figure 12C).

## 4. Discussion

Early intestinal microbiota establishment is crucial for intestinal physiology and regulation throughout adult life. Our previous studies found significant alterations in the small intestinal and colonic microbiome and metabolome profiles of IUGR piglets during the suckling and weaning stages [15,17]. However, the effects of IUGR on colonic microbiota colonization and metabolism in pigs during the growing-finishing stages remained unclear. The present study investigated the impacts of IUGR on plasma biochemical parameters and colonic microbiota community, metabolite profiles, and barrier function in growing-finishing pigs. We found that IUGR affected lipid metabolism and colonic barrier function by reducing antioxidant capacity *via* the Nrf2/Keap1 pathway, as well as activating colonic inflammation *via* the TLR4-NF-κB/ERK pathway in growing-finishing pigs.

Plasma biochemical parameters reflect animals’ physiological, nutritional, and pathological status. Plasma ALB and TP concentrations are indicators of the utilization efficiency of dietary protein in pigs, and the increase in plasma UN concentration indicates a reduction in the protein utilization rate [18]. In the present study, IUGR decreased plasma TP and ALB concentrations while increasing plasma UN in pigs, suggesting that IUGR decreased the protein utilization efficiency from diets and led to a deficiency in protein anabolism, consistent with a previous study [19]. Those alterations in plasma may be associated with impaired intestinal amino acid absorption and utilization rates in IUGR pigs [20]. Furthermore, IUGR pigs showed a lower plasma GLU level at the 25 and 50 kg BW stages. Previous studies indicated that IUGR could lead to lower dietary starch digestibility and glucose absorption throughout life, resulting in a lower plasma GLU level [21,22,23]. Therefore, we postulated that IUGR pigs might have a lower intestinal glucose absorption rate.

Intestinal epithelial function mainly depends on tight junctions (TJs), including occludin, claudins, and ZO-1 barrier proteins [24]. As a physical barrier function, intestinal TJs play primary roles in maintaining intercellular interactions and stabilizing paracellular and transcellular pathways [25]. ZO-1 is a peripheral membrane scaffolding protein associated with the distribution and maintenance of TJs [26]. Occludin is devoted to the transfer of macromolecular substances through the cellular bypass pathway by activating directly with claudins and actin [27]. Claudins are composed of multiple families, and some proteins have sealing functions (including claudins 1, 3, 5, 11, 14, and 19). In contrast, a significant number of claudins form channels across TJs that feature selectivity for cations (including claudins 2, 10b, and 15), anions (including claudin-10a and 17), or are permeable to water (claudin-2) [28]. In the present study, occludin, ZO-1, and claudin-1 abundances were significantly reduced in IUGR pigs, which might be a hint for a disturbed barrier function. We speculated that other members of the claudins family could be involved in that dysfunction. Previous studies demonstrated that IUGR impaired intestinal epithelial TJs (e.g., ZO-1 and occludin) [29]; moreover, occludin and claudin-1 abundances were reduced in the colon of IUGR pigs at the growing stage [30]. To date, the effects of IUGR on the Claudins family, except the claudin-1, is still unclear, which warrants further study.

It is worth noting that the damage caused by IUGR in colonic barrier function is not limited to infancy and childhood but spans adulthood. However, our results showed that the long-term adverse effects persisted in barrier function and a lessened disparity in TJs proteins between the NBW and IUGR pigs, which might be associated with the catch-up growth. Another study revealed that IUGR piglets with a catch-up growth before weaning exhibited a recovered intestinal physical barrier, including occludin, claudins, and ZO-1, almost as good as NBW piglets [31]. The “thrifty phenotype” hypothesis suggests that when nutritional conditions in the uterus are suboptimal, metabolism and growth of the fetus are restricted; but when the postnatal nutritional condition is adequate, IUGR pigs undergo a catch-up growth, such a process is likely resulting from an adaptive process in adverse conditions [1,14]. Hence, we speculated that a catch-up growth-associated intestinal barrier damage recovery occurred at the growing-finishing stage in the IUGR pigs.

To explore whether IUGR-induced colonic barrier damage was associated with oxidative and inflammatory pathways, the oxidative Nrf2/Keap1 and inflammatory TLR4-NFκB/ERK pathways were evaluated. Mammalian possesses several redox defense systems, including SOD, GPX, and GSH [32]. IUGR predisposes newborns and youth to oxidative imbalance and inflammation, and the effect lasts for a long time in adult life. Under physiological conditions, Nrf2 binds to Keap1 in the cytoplasm [33]. To combat the reactive oxygen species (ROS) stress, the isolated Keap1/Nrf2 complex urges the phosphorylation of Nrf2 to translocate into the nucleus and activate the transcription of antioxidant genes [34]. In the present study, IUGR reduced the antioxidant capacity parameters such as SOD, GSH, and GPX in the colon of growing-finishing pigs by inhibiting the phosphorylated Nrf2 and facilitating Keap1 activity. Recent research also showed that IUGR decreased SOD activity, GSH, and GPX levels in the small intestine and restrained the classical Nrf2/Keap1 oxidative stress defense system in weaned pigs [34,35]. A previous study reported that Nrf2-mediated oxidative stress and inflammation may indirectly promote intestinal TJ function [36]. These findings suggest that the redox imbalance might be the reason why the colonic barrier damage appeared in IUGR pigs.

The increased ROS causes damage in the gut, resulting in pathogenic invasions, which release LPS and stimulate inflammatory responses [37]. LPS stimulates TLR4 and subsequently recruits MyD88 [38], which recruits the transforming growth factor β (TGF-β)-activated kinase 1 (TAK1), resulting in the IκB-α kinase complex activation. The NF-κB protein is suppressed by inhibitors of IκB binding in the cytoplasm [39]. Subsequently, the IκB-α kinase phosphorylated IκB-α protein, which allows NF-κB to translocate to the nucleus, and it also facilitates the transcription of the proinflammatory cytokines (including IL-1β and TNF-α) to affect the intestinal barrier integrity [40]. In contrast, IL-10, as an anti-inflammatory cytokine, antagonizes the effects caused by the proinflammatory cytokines on the TJ proteins [41]. TLR4 also activates the downstream mitogen-activated protein kinases (MAPK) pathway, and TAK1 is an essential intermediate for activating MAPK cascades [42]. TAK1 activates MAPK kinases (MAPKK), which in turn phosphorylates three MAPKs, including the extracellular signal-regulated kinase1/2 (ERK1/2) [39]. Moreover, Tao et al. [29] also reported that IUGR deteriorated the hindgut barrier (ZO-1 and occludin) and increased the mucosal *IL-1β* and *TNF-α* expressions in pigs at the growing stage. Another recent study found that IUGR impaired intestinal morphology and increased inflammation by activating the TLR4/NF-κB pathway in weaned piglets [43]. Our results showed that IUGR up-regulated colonic IL-1β and TNF-α expressions, down-regulated IL-10 expression, and up-regulated relative protein abundances of TLR4-NF-κB/ERK pathway in growing-finishing pigs. Therefore, we speculated that IUGR impaired epithelial function, and the invasion of LPS-producing bacteria became easier and further induced inflammation through activating the TLR4-NF-κB/ERK pathway in growing-finishing pigs.

The mammalian intestine is the harbor of microbiota, and the microbial alpha diversity is considered a marker of gut homeostasis [44]. Our results showed that the IUGR pigs had a higher Simpson index at the 50 kg BW stage. Huang et al. [30] also reported that IUGR pigs had higher alpha diversity in the ileum than the NBW pigs at 70 days old. At the phylum level, Firmicutes and Bacteroidetes were the top two most abundant phyla in the IUGR pigs throughout the trial, consistent with a previous study [45]. In addition, IUGR pigs had a lower F/B ratio at the 25 kg BW stage but higher Firmicutes abundance at the 100 kg BW stage. The higher Firmicutes abundance is related to energy intake from diets [46], and body fat deposition is associated with Firmicutes abundance and the F/B ratio in the intestine [47]. These findings suggest that higher Firmicutes abundance contributed to lipid absorption and deposition in IUGR pigs during the finishing stage, which is in accordance with the higher plasma TG and CHO levels, as mentioned above in the present study.

*Lactobacillus* and *Streptococcus* were the predominant colonic microbiota in IUGR pigs in the present study, which is consistent with a previous study [43]. *Streptococcus* is composed of several opportunistic pathogens [48]. The lower *Streptococcus* abundance in the colon at the 25 kg BW stage suggests that an impaired redox status in IUGR pigs is independent of the microbial barrier. *Streptococcus* is also known as a bioamine producer [49] and is positively correlated with phenylethylamine and 1,7-heptyldiamine. Lactic acid-producing bacteria *Lactobacillus* could degrade lactose into acetate [50]. However, we found that *Lactobacillus* was positively correlated with spermine and tyramine but had no correlation with SCFAs. Although some bacterial genera were correlated with SCFAs and bioamines, the possible reason might be the microbial interactions, such as resource competition; however, it is still difficult to ensure which microbes related to the production of specific colonic metabolites and warrant further studies [51].

The SCFAs, especially butyrate, provide 60–70% of the total energy to the colonic epithelial cells and ∼10% of the daily caloric requirements [52]. We found that IUGR pigs had lower colonic concentrations of butyrate, valerate, and acetate, which might be related to the decreased SCFAs-producing bacteria, such as *Lactobacillus* and *unclassified_Lachnospiraceae*. Moreover, Spearman’s correlation revealed a positive correlation between acetate and valerate with *Lactobacillus* and *unclassified_Lachnospiraceae* in the colon. Based on these findings, we postulated that decreased colonic SCFA concentrations in IUGR pigs might lead to a reduced energy source salvaged from undigested carbohydrates and proteins for animals. The lower fermentation energy combined with those mentioned earlier destroyed intestinal physiological status; thereby, IUGR affected the growth performance of pigs in our previous study [14].

The increased colonic bioamines (such as cadaverine and putrescine), phenol, and skatole are toxic to gut health and cause diarrhea in pigs [53]. Our findings showed that colonic cadaverine concentration was increased in the IUGR pigs at the 25 kg BW stage. Moreover, colonic putrescine concentration at the 50 and 100 kg BW stages and cadaverine concentration at the 100 kg BW stage were lower in the IUGR pigs. The gastrointestinal dysfunction of IUGR pigs might explain this discrepancy. Oxidative stress resulting from bioamine catabolism is considered to damage DNA and proteins [54]. IUGR pigs had a higher gene function related to the cancer pathway at the 25 kg BW stage, suggesting that IUGR may lead to impairment in colonic epithelial cells at the early growth stage. The enriched cancer pathway might be related to the excessive bioamine concentrations in the colon of IUGR pigs.

Identification and quantification of compounds in the metabolome can be used to define the metabolic changes associated with physiological differences and external disturbances [55]. In the present study, the most enriched differential metabolites included lipids and lipid-like molecules, organic acids and derivatives, and organoheterocyclic compounds, which were noteworthy for discussion. IUGR increased 14 differential metabolites from lipids and lipid-like molecules (e.g., sterol, 3-oxooctadecanoic acid, PC, and others), suggesting a potential dysfunction in lipid biosynthesis and metabolism in the colon of IUGR pigs. Specifically, excessive sterols and cholesterol cause cardiovascular disorders (such as hypercholesterolemia) and several congenital diseases [56]. 3-oxooctadecanoic acid, converted from malonic acid via the enzyme, is an intermediate in fatty acid biosynthesis. Excessive changes in the plasma PC and/or PE contents and intestinal metabolites are implicated in metabolic disorders, such as insulin resistance and obesity [57]. It has been reported that IUGR altered several metabolites associated with lipogenesis in fetal [58], neonatal [13], and growing pigs [30]. Previous studies reported that IUGR pigs are most likely to develop metabolic and cardiovascular disorders due to abnormal fat storage and lipid metabolism in adulthood [30]. Our findings suggest that the excessively higher concentrations of sterols, PC, and PE might be relevant to the risk of cardiovascular disorders in IUGR pigs. In other words, the alterations of these metabolites may contribute to abnormal lipid metabolism in IUGR pigs.

In addition, nine organoheterocyclic compounds in the colonic contents of IUGR pigs (e.g., pyridoxic acid, adenine, and cytosine) were higher than those in the NBW pigs. 4-pyridoxic acid is the catabolic product of vitamin B_6_, which can be further broken down by the gut microbiota via 4-pyridoxic acid dehydrogenase [59]. A higher pyridoxic acid concentration might show a lack of this enzyme in IUGR pigs. The concentrations of eight differential metabolites increased in colonic contents of IUGR pigs from organic acids and derivatives (e.g., methionyl-proline and isoleucyl-tryptophan), which are incomplete catabolic dipeptides of protein digestion or proteolysis [60]. The enrichments of these metabolites in the colonic contents of IUGR pigs indicate a reduction in complete protein breakdown efficiency in the gut. The present study also showed that IUGR pigs had relatively higher incomplete breakdown products (dipeptides) and lower complete breakdown products (amino acids) in the colon, further confirmed by the increased bioamines in the colon at the 100 kg BW stage.

Furthermore, based on metabolic pathway analysis, three differential metabolites (including 12,13-EpOME, phytosphingosine, and choline) enriched the four metabolic pathways related to lipid metabolism at the 25 kg BW stage. The enrichment of these pathways might be associated with abnormal lipid metabolism in IUGR pigs. In the present study, the metabolic changes were paralleled by intestinal microbiota alterations. Moreover, *Mogibacteriaceae* abundance was positively correlated with choline, N-a-acetyl-L-arginine, O-propanoyl-carnitine, squamolone, and PE (P-16:0/14:0) at the 25 kg BW stage in IUGR pigs, whereas it was negatively correlated with 25 metabolites and pathway enrichment at the 100 kg BW stage in NBW pigs. Furthermore, all these metabolites were increased in IUGR pigs at the 25 and 100 kg BW stages, and the change trends of these results were consistent. Collectively, the turbulence of the colonic microbial community and metabolic homeostasis could be the main underlying factor leading to the stunted growth performance of IUGR pigs during the growing-finishing stage.

## 5. Conclusions

In summary, IUGR continued to disrupt colonic barrier function by inhibiting antioxidant capacity *via* the Nrf2/Keap1 pathway and activating inflammation *via* the TLR4-NF-κB/ERK pathway in growing-finishing pigs. Moreover, IUGR pigs exhibited suboptimal lipid metabolism. Notably, the increased colonic concentrations of organic acids and derivatives, lipids and lipid-like molecules, and dipeptides may be linked to the above-mentioned metabolic disorders in IUGR pigs. The alterations of Firmicutes and *Streptococcus* abundances might be associated with nutrient absorption and colonic health of IUGR pigs.

## Figures and Tables

**Table 1:** Effects of IUGR on plasma biochemical parameters in growing-finishing pigs.

Items	25 kg BW Stage		50 kg BW Stage		100 kg BW Stage	
	NBW	IUGR	NBW	IUGR	NBW	IUGR
ALB (g/L)	34.33 ± 1.07	33.80 ± 1.46	41.33 ± 1.32	35.63 ± 1.38 *	52.39 ± 0.89	47.74 ± 1.54 *
AMM (μmol/L)	171.90 ± 15.90	157.13 ± 3.93	164.17 ± 22.40	234.97 ± 23.29 *	159.02 ± 17.19	206 ± 23.63
ALT (U/L)	41.00 ± 1.90	45.66 ± 2.05	31.42 ± 1.68	37.66 ± 1.48 *	41.42 ± 2.01	38.77 ± 2.05
AST (U/L)	68.29 ± 4.09	80.71 ± 5.22	65.50 ± 4.63	73.33 ± 5.79	63.92 ± 4.94	75.64 ± 13.31
ALP (U/L)	256.00 ± 7.74	298.86 ± 3.94 *	170.42 ± 8.83	177.25 ± 9.86	138.08 ± 7.62	138.82 ± 7.30
CHO (mmol/L)	2.60 ± 0.02	2.37 ± 0.12	2.26 ± 0.06	2.48 ± 0.10 *	2.48 ± 0.07	2.58 ± 0.05
CHE (U/L)	600.00 ± 27.93	598.57 ± 21.76	717.67 ± 27.08	630.42 ± 23.26 *	585.17 ± 26.63	606.27 ± 34.66
GLB (g/L)	21.60 ± 1.17	21.91 ± 0.95	26.53 ± 1.33	28.46 ± 1.96	26.36 ± 2.10	25.26 ± 2.03
GLU (mmol/L)	6.60 ± 0.10	4.94 ± 0.20 *	6.17 ± 0.24	5.33 ± 0.29 *	5.83 ± 0.30	5.80 ± 0.71
HDL-C (mmol/L)	0.94 ± 0.03	1.05 ± 0.05	0.93 ± 0.04	0.98 ± 0.04	1.15 ± 0.06	1.07 ± 0.05
LDL-C (mmol/L)	1.42 ± 0.01	1.50 ± 0.07	1.17 ± 0.05	1.20 ± 0.06	1.36 ± 0.04	1.41 ± 0.04
TG (mmol/L)	0.53 ± 0.01	0.57 ± 0.01	0.46 ± 0.03	0.57 ± 0.04 *	0.61 ± 0.05	0.67 ± 0.07
TP (g/L)	55.93 ± 1.63	55.71 ± 0.71	67.86 ± 1.19	64.09 ± 0.94 *	78.75 ± 1.87	73.00 ± 1.89 *
UN (mmol/L)	2.34 ± 0.21	2.03 ± 0.09	4.28 ± 0.34	3.72 ± 0.36	6.64 ± 0.34	7.65 ± 0.30 *

Data are presented as means ± SEM (*n* = 10). * *p* < 0.05. IUGR, intrauterine growth restriction; NBW, normal birth weight; ALB, albumin; ALT, alanine aminotransferase; ALP, alkaline phosphatase; AMM, ammonia; AST, aspartate aminotransferase; CHE, cholinesterase; CHO, cholesterol; GLB, globulin; GLU, glucose; HDL-C, high-density lipoprotein-cholesterol; LDL-C, low-density lipoprotein-cholesterol; TG, triglyceride; TP, total protein; UN, urea nitrogen.

**Table 2:** Effects of IUGR on the relative abundances of colonic microbiota communities in growing-finishing pigs.

Items (%)	25 kg BW Stage		50 kg BW Stage		100 kg BW Stage	
	NBW	IUGR	NBW	IUGR	NBW	IUGR
Firmicutes	86.11 ± 2.74	78.93 ± 4.38	84.89 ± 5.49	92.35 ± 1.51	91.41 ± 1.49	95.87 ± 0.66 *
Bacteroidetes	9.38 ± 2.35	17.47 ± 4.21	13.70 ± 0.05	5.22 ± 0.61	4.45 ± 1.49	2.89 ± 0.62
F/B	13.58 ± 3.53	6.21 ± 1.72 *	19.14 ± 3.14	40.79 ± 6.35	37.57 ± 6.58	35.50 ± 5.45
Lactobacillaceae	27.25 ± 4.28	64.74 ± 8.50 *	53.39 ± 6.48	55.18 ± 7.46	22.63 ± 3.86	27.45 ± 4.83
*Streptococcus*	32.23 ± 5.62	16.82 ± 3.71 *	7.54 ± 0.04	6.99 ± 0.03	18.45 ± 2.54	17.98 ± 3.55
*Lactobacillus*	21.23 ± 2.67	20.56 ± 2.54	53.39 ± 4.75	55.18 ± 5.45	26.86 ± 3.30	22.34 ± 3.42
*unclassified*_*Lachnospiraceae*	1.60 ± 0.25	2.27 ± 0.47	3.62 ± 0.08	6.99 ± 0.18	25.21 ± 4.35	16.27 ± 3.68

Data are presented as means ± SEM (*n* = 10). * *p* < 0.05. F/B, Firmicutes-to-Bacteroidetes ratio; IUGR, intrauterine growth restriction; NBW, normal birth weight.

**Table 3:** Effects of IUGR on colonic short-chain fatty acids concentration in growing-finishing pigs.

Items (mg/g)	25 kg BW Stage		50 kg BW Stage		100 kg BW Stage	
	NBW	IUGR	NBW	IUGR	NBW	IUGR
Acetate	3.29 ± 0.08	3.21 ± 0.32	4.79 ± 0.27	4.66 ± 0.24	5.12 ± 0.17	4.40 ± 0.17 *
Propionate	1.49 ± 0.03	1.47 ± 0.19	1.81 ± 0.08	1.70 ± 0.10	1.81 ± 0.16	1.75 ± 0.14
Isobutyrate	0.19 ± 0.02	0.14 ± 0.01 *	0.20 ± 0.04	0.23 ± 0.03	0.28 ± 0.02	0.19 ± 0.02 *
Butyrate	1.11 ± 0.06	0.90 ± 0.07 *	1.57 ± 0.13	1.19 ± 0.11 *	1.22 ± 0.09	1.20 ± 0.14
Isovalerate	0.30 ± 0.03	0.22 ± 0.02 *	0.34 ± 0.08	0.37 ± 0.05	0.48 ± 0.04	0.33 ± 0.04 *
Valerate	0.28 ± 0.04	0.25 ± 0.03	0.51 ± 0.06	0.29 ± 0.03 *	0.31 ± 0.01	0.28 ± 0.04
SCFAs	6.17 ± 0.11	5.84 ± 0.52	8.50 ± 0.36	7.99 ± 0.39	8.20 ± 0.42	7.79 ± 0.41
BCFAs	0.49 ± 0.04	0.36 ± 0.02 *	0.54 ± 0.12	0.61 ± 0.08	0.76 ± 0.06	0.52 ± 0.06 *
SCFAs + BCFAs	6.62 ± 0.12	6.20 ± 0.53	9.04 ± 0.46	8.60 ± 0.45	8.96 ± 0.39	8.39 ± 0.44

Data are presented as means ± SEM (*n* = 12). * *p* < 0.05. IUGR, intrauterine growth restriction; NBW, normal birth weight; SCFAs, short-chain fatty acids (including acetate, butyrate, propionate, and valerate); BCFAs, branched-chain fatty acids (including isobutyrate and isovalerate).

**Table 4:** Effects of IUGR on colonic indole, skatole, and bioamine concentrations in growing-finishing pigs.

Items (mg/g)	25 kg BW Stage		50 kg BW Stage		100 kg BW Stage	
	NBW	IUGR	NBW	IUGR	NBW	IUGR
1,7-heptyl diamine	0.22 ± 0.07	0.11 ± 0.01	0.14 ± 0.03	0.11 ± 0.02	0.16 ± 0.06	0.08 ± 0.01
Cadaverine	3.65 ± 0.70	6.65 ± 0.53 *	2.96 ± 0.91	2.44 ± 0.84	1.46 ± 0.22	0.72 ± 0.18 *
Indole	4.58 ± 1.31	6.56 ± 2.43	7.18 ± 1.52	2.41 ± 0.59 *	11.15 ± 1.28	7.24 ± 0.92 *
Phenylethylamine	0.13 ± 0.04	0.09 ± 0.03	0.10 ± 0.02	0.08 ± 0.02	0.10 ± 0.04	0.04 ± 0.01
Putrescine	2.46 ± 0.41	2.62 ± 0.36	4.00 ± 0.67	2.03 ± 0.40 *	1.36 ± 0.23	0.69 ± 0.16 *
Skatole	13.20 ± 3.20	10.97 ± 2.3	18.21 ± 2.16	17.63 ± 4.55	18.08 ± 3.44	21.29 ± 5.81
Spermidine	3.33 ± 0.74	2.29 ± 0.27	2.60 ± 0.41	2.34 ± 0.35	1.50 ± 0.22	1.24 ± 0.15
Spermine	0.53 ± 0.12	0.47 ± 0.08	0.47 ± 0.07	0.37 ± 0.04	0.18 ± 0.02	0.16 ± 0.02
Tryptamine	1.03 ± 0.22	0.37 ± 0.12	0.41 ± 0.12	0.24 ± 0.06	0.23 ± 0.06	0.16 ± 0.05
Tyramine	1.56 ± 0.32	1.54 ± 0.31	0.48 ± 0.17	0.58 ± 0.19	1.13 ± 0.33	0.74 ± 0.19
Total bioamine	13.85 ± 2.72	13.81 ± 1.22	11.50 ± 1.71	8.70 ± 1.82	5.95 ± 1.04	3.61 ± 0.49

Data are presented as means ± SEM (*n* = 12). * *p* < 0.05. IUGR, intrauterine growth restriction; NBW, normal birth weight.

**Table 5:** Metabolic pathways and significantly differential metabolite markers between IUGR and NBW pigs during the growing-finishing stage.

Pathways	*p*-Values	Impact	Matched Significantly Differential Metabolites
25 kg BW stage			
Linoleic acid metabolism	0.011	0	12,13-EpOME
Sphingolipid metabolism	0.045	0	Phytosphingosine
Glycerophospholipid metabolism	0.061	0.024	Choline
Glycine, serine, and threonine metabolism	0.067	0	Choline
100 kg BW stage			
Purine metabolism	0.001	0.027	Deoxyadenosine; guanine; Deoxyguanosine; guanosine; adenine
Pyrimidine metabolism	0.065	0.010	Deoxycytidine; 3-aminoisobutanoic acid
Vitamin B_6_ metabolism	0.099	0	4-pyridoxic acid
Pentose phosphate pathway	0.198	0	Deoxyribose

BW, body weight; IUGR, intrauterine growth restriction; NBW, normal birth weight.

## Data Availability

The data analyzed during the current study are available from the corresponding author upon reasonable request.

## Supplementary Materials

The following supporting information can be downloaded at https://www.mdpi.com/article/10.3390/antiox13030283/s1, Figure S1: Rarefaction curve analysis was used to evaluate whether further sequencing would likely detect additional taxa; Figure S2: Correlations between colonic SCFAs, indole, skatole, and bioamines concentrations and the relative abundances of microbial genera at the 25 (A), 50 (B), and 100 (C) kg body weight (BW) stages; Table S1: Ingredients and chemical composition of the experimental diets (as-fed basis); Table S2: Primer sequences used in the RT-PCR.
