# Supplementary material for: Intrauterine Growth Restriction Affects Colonic Barrier Function via Regulating the Nrf2/Keap1 and TLR4-NF-κB/ERK Pathways and Altering Colonic Microbiome and Metabolome Homeostasis in Growing–Finishing Pigs
Source: Antioxidants (Basel). 2024 Feb 26;13(3):283. doi: 10.3390/antiox13030283 (PMC10967500; doi:10.3390/antiox13030283)
Supplement: Supplementary file 1 [file antioxidants-13-00283-s001.zip › antioxidants-2816187-supplementary.pdf]

antioxidants

MDPI

Article

Intrauterine Growth Restriction Affects Colonic Barrier Function via Regulating the Nrf2/Keap1 and TLR4-NF-κB/ERK Pathways and Altering Colonic Microbiome and Metabolome Homeostasis in Growing–Finishing Pigs

Liang Xiong [1,2], Md. Abul Kalam Azad [1], Yang Liu [1], Wanghong Zhang [1], Qian Zhu [1], Chengjun Hu [1], Jinming You [2,*] and Xiangfeng Kong [1,*]

[1] Key Laboratory of Agro-Ecological Processes in Subtropical Region, Hunan Provincial Key Laboratory of Animal Nutritional Physiology and Metabolic Process, National Engineering Laboratory for Pollution Control and Waste Utilization in Livestock and Poultry Production, Institute of Subtropical Agriculture, Chinese Academy of Sciences, Changsha 410125, China; 244628426@stu.scau.edu.cn (L.X.); azadmak@isa.ac.cn (M.A.K.A.); liuyang@stu.njau.edu.cn (Y.L.); 13616905519@stu.scau.edu.cn (W.Z.); zhuqian@isa.ac.cn (Q.Z.); yxh@stu.scau.edu.cn (C.H.)

[2] Key Laboratory of Animal Nutrition in Jiangxi Province, College of Animal Science and Technology, Jiangxi Agricultural University, Nanchang 330045, China

* Correspondence: youjinm@jxau.edu.cn or youjinm@163.com (J.Y.); nnkxf@isa.ac.cn (X.K.)

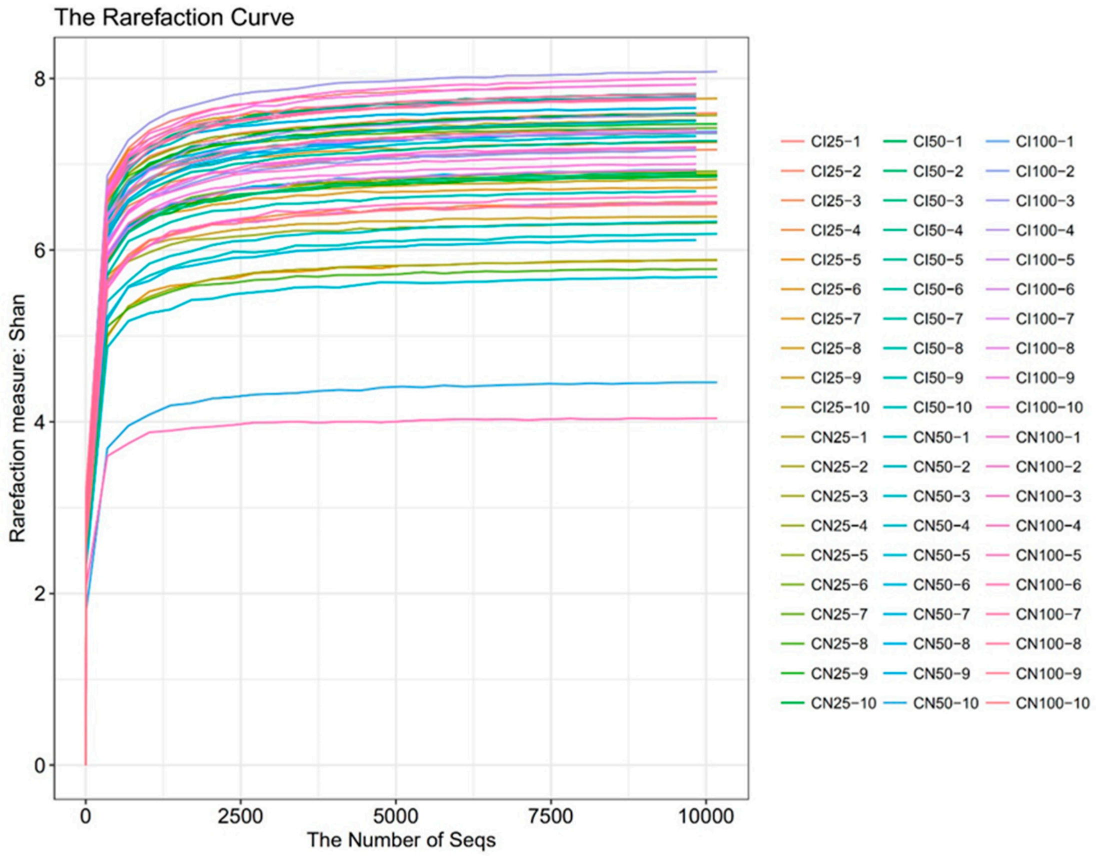

Figure S1. Rarefaction curve analysis was used to evaluate whether further sequencing would likely detect additional taxa. CI and CN represent samples obtained from the colon contents of intrauterine growth restriction (IUGR) pigs and normal birth weight (NBW) pigs, respectively; 25, 50, and 100 represent 25, 50, and 100 kg body weight stages of NBW pigs, respectively.

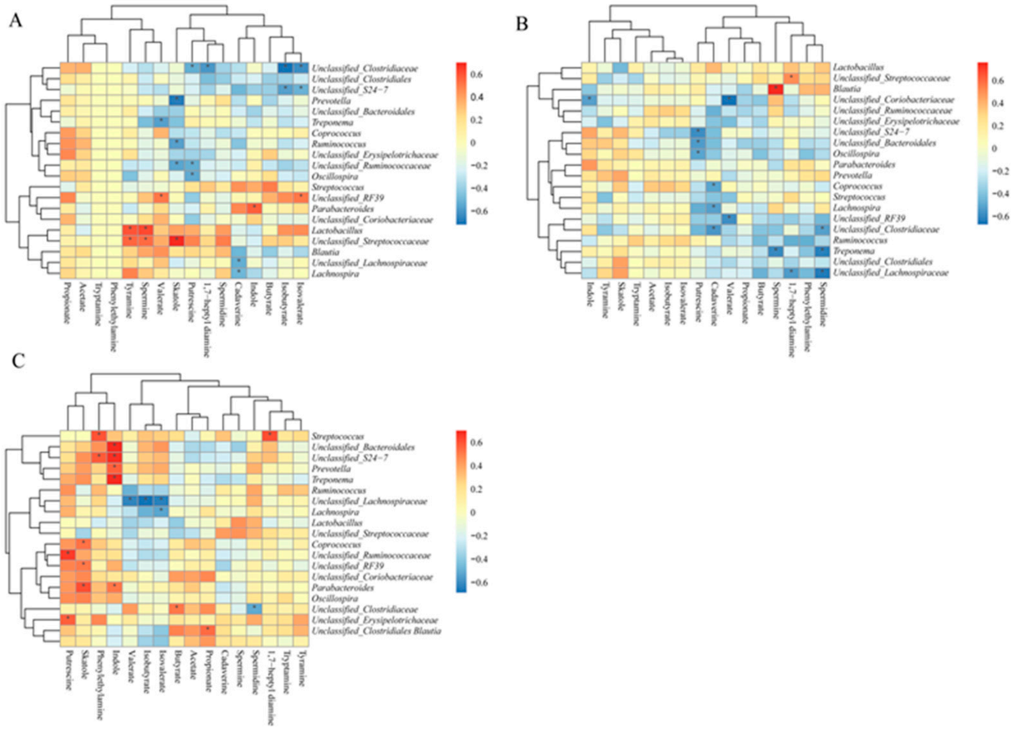

Figure S2. Correlations between colonic SCFAs, indole, skatole, and bioamines concentrations and the relative abundances of microbial genera at the 25 (A), 50 (B), and 100 (C) kg body weight (BW) stages. Cells are colored based upon the Spearman's correlation coefficient between the microbial genera and colonic metabolites. The red, blue, and white represent significant positive correlations, negative correlations, and no significant correlation, respectively. * $P < 0.05$.

Table S1 Ingredients and chemical composition of the experimental diets (as-fed basis).

Items	Nursery pig feed (28-69 day-old)	Growing pig feed (70-103 day-old)	Finishing pig feed (104-165 day-old)
Ingredients (%)			
Corn	60.00	61.00	61.17
Soybean meal	27.50	25.00	25.50
Barley	6.00	8.00	8.00
Soybean oil	2.00	1.50	1.00
Lysine	0.16	0.18	0.13
$CaHPO_4$	0.10	0.10	0.00
Threonine	0.10	0.07	0.08
Methionine	0.02	0.03	0.00
Anti-mildew agent	0.10	0.10	0.10
Anti-oxidant	0.02	0.02	0.02
Nursery pigs premix[1)]	4.00	0.00	0.00
Growing-finishing pigs premix[2)]	0.00	4.00	4.00
Total	100.00	100.00	100.00
Nutrient levels[3)]			

Digestible energy (MJ/kg)	13.91	13.77	13.64
Crude protein	17.20	16.40	16.50
Crude fat	4.70	4.30	3.80
Crude fiber	2.70	2.70	2.80
Digestible lysine	1.17	1.08	1.05
Digestible methionine	0.33	0.30	0.28
Digestible threonine	0.77	0.71	0.73
Total calcium	0.77	0.74	0.66
Total phosphorus	0.56	0.52	0.45

[1)] The nursery pig premix supplied for per kg diet: vitamin A 8 000 IU, vitamin D_3 228 IU, vitamin E 15 IU; vitamin K_3 3.00 mg, vitamin B_1 1.30 mg, vitamin B_2 3.10 mg, vitamin B_6 1.20 mg, vitamin B_{12} 0.03 mg, calcium pantothenate 13.40 mg, choline chloride 500 mg, iron 120 mg, copper 10 mg, zinc 130 mg, manganese 100 mg, iodine 0.30 mg, and selenium 0.30 mg.

[2)] The growing-finishing pig premix supplied for per kg diet: vitamin A 15 000 IU, vitamin D_3 200 IU, vitamin E 50 IU, vitamin K_3 4.00 mg, vitamin B_1 4.00 mg, vitamin B_2 10 mg, vitamin B_6 3.00 mg, vitamin B_{12} 0.04 mg, calcium pantothenate 20.00 mg, choline chloride 800 mg, iron 120 mg, copper 20 mg, zinc 112 mg, manganese 124 mg, iodine 0.50 mg, and selenium 0.40 mg.

[3)] Nutrient levels were calculated values.

Table S2 Primer sequences used in the RT-PCR.

Target genes	Primers	Sequences (5′-3′)	Product size (bp)
β-actin	Forward	GATCTGGCACCACACCTTCTACAAC	107
	Reverse	TCATCTTCTCACGGTTGGCTTTGG	
GPX1	Forward	TGGGGAGATCCTGAATT	184
	Reverse	GATAAACTTGGGGTCGG	
GPX4	Forward	GATTCTGGCCTTCCCTTGC	173
	Reverse	TCCCCTTGGGCTGGACTTT	
SOD1	Forward	GAGACCTGGGCAATGTGACT	189
	Reverse	CCAAACGACTTCCAGCATTT	
SOD2	Forward	TGTATCCGTCGGCGTCCAAGG	93
	Reverse	TCCTGGTTAGAACAAGCGGCAATC	
IL-1β	Forward	ACCTGGACCTTGGTTCTC	124
	Reverse	GGATTCTTCATCGGCTTC	
IL-10	Forward	CACTGCTCTATTGCCTGATCTTCC	136
	Reverse	AAACTCTTCACTGGGCCGAAG	
TNF-α	Forward	ACGCTCTTCTGCCTACTGC	162
	Reverse	TCCCTCGGCTTTGACATT	

GPX, glutathione peroxidase; *SOD*, superoxide dismutase; *IL*, interleukin; *TNF-α*, tumor necrosis factor α.

Disclaimer/Publisher's Note: The statements, opinions and data contained in all publications are solely those of the individual author(s) and contributor(s) and not of MDPI and/or the editor(s). MDPI and/or the editor(s) disclaim responsibility for any injury to people or property resulting from any ideas, methods, instructions or products referred to in the content.
